# Titanium-Dioxide-Nanoparticle-Embedded Polyelectrolyte Multilayer as an Osteoconductive and Antimicrobial Surface Coating

**DOI:** 10.3390/ma16217026

**Published:** 2023-11-03

**Authors:** Matthew Rothpan, Nitin Chandra Teja Dadi, Geoffrey McKay, Michael Tanzer, Dao Nguyen, Adam Hart, Maryam Tabrizian

**Affiliations:** 1 Department of Biomedical Engineering, Faculty of Medicine and Health Sciences, McGill University, Montreal, QC H3A 2B6, Canada; matthew.rothpan@mail.mcgill.ca; 2 Jo Miller Orthopaedic Research Laboratory, Division of Orthopaedic Surgery, McGill University, Montreal, QC H3G 1A4, Canada; nitin.dadi@mail.mcgill.ca (N.C.T.D.); michael.tanzer@mcgill.ca (M.T.); 3 Meakins-Christie Laboratories, Research Institute of the McGill University Health Centre, Montreal, QC H4A 3J1, Canada; geoffrey.mckay@affiliate.mcgill.ca (G.M.); dao.nguyen@mcgill.ca (D.N.); 4 Department of Microbiology and Immunology, McGill University, Montreal, QC H3A OG4, Canada; 5 Department of Medicine, McGill University, Montreal, QC H4A 3J1, Canada; 6 Faculty of Dentistry and Oral Health Sciences, McGill University, 3640 Rue University, Montreal, QC H3A 0C7, Canada

**Keywords:** titanium dioxide nanoparticles, silver nanoparticles, polyelectrolyte, layer by layer, antibiofilm coating, bioactive coating, orthopedic implants

## Abstract

Bioactive surface coatings have retained the attention of researchers and physicians due to their versatility and range of applications in orthopedics, particularly in infection prevention. Antibacterial metal nanoparticles (mNPs) are a promising therapeutic, with vast application opportunities on orthopedic implants. The current research aimed to construct a polyelectrolyte multilayer on a highly porous titanium implant using alternating thin film coatings of chitosan and alginate via the layer-by-layer (LbL) self-assembly technique, along with the incorporation of silver nanoparticles (AgNPs) or titanium dioxide nanoparticles (TiO_2_NPs), for antibacterial and osteoconductive activity. These mNPs were characterized for their physicochemical properties using quartz crystal microgravimetry with a dissipation system, nanoparticle tracking analysis, scanning electron microscopy, and atomic force microscopy. Their cytotoxicity and osteogenic differentiation capabilities were assessed using AlamarBlue and alkaline phosphatase (ALP) activity assays, respectively. The antibiofilm efficacy of the mNPs was tested against *Staphylococcus aureus*. The LbL polyelectrolyte coating was successfully applied to the porous titanium substrate. A dose-dependent relationship between nanoparticle concentration and ALP as well as antibacterial effects was observed. TiO_2_NP samples were also less cytotoxic than their AgNP counterparts, although similarly antimicrobial. Together, these data serve as a proof-of-concept for a novel coating approach for orthopedic implants with antimicrobial and osteoconductive properties.

## 1. Introduction

**Figure 1:**
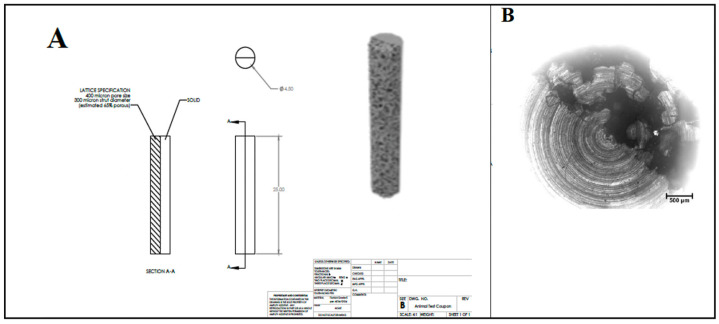
Details of macrostructure (**A**) and microstructure (**B**) of titanium substrates from which the disk samples were cut.

Hip and knee replacements cost Canada CAD 1.3 billion and were performed on 1.3 million people in 2020–2021 [1]. Treatment requires multiple surgeries, wound debridement, and implant removal. Prolonged intravenous antibiotic therapy is also required. However, infection eradication rates are only 83–87% [2]. Patients with periprosthetic joint infection (PJI) have longer hospital stays, higher costs, and more time in the operating room. This results in an annual cost of over CAD 42 million and 25,349 days of hospitalization. In the USA, hospital expenses for PJI will reach USD 1.85 billion by 2030 for hip and knee procedures [3]. PJI is increasingly problematic due to the growing antimicrobial resistance in the microorganisms that are primarily responsible for implant failure following the joint replacement surgery (Figure 1) [4,5,6,7,8].

Gram-positive Staphylococci biofilms account for approximately 75% of PJI, as biofilm increases resistance to antibiotics 500–5000 times more than planktonic cells [9,10,11]. Additionally, owing to its high adaptability and frequent exposure to antibiotic therapies, *Staphylococcus aureus* has undergone inadvertent selection for drug-resistant strains, leading to therapeutic failures [12,13,14,15]. The conventional treatments often require multiple surgeries and extended antibiotic therapy, resulting in high morbidity and poor patient outcomes [7,16,17,18,19,20,21].

In this context, the most common approach involves the use of metal nanoparticles (mNPs) such as silver [22,23,24,25], zinc [26,27,28,29], copper [30,31,32,33,34], gold [35,36,37,38], or titanium dioxide [39,40,41,42]. Their attractive antimicrobial properties arise from metal ion release (bactericidal), oxidative stress, and non-oxidative mechanisms (Table 1) [43,44,45,46,47,48,49]. Silver nanoparticles (AgNPs) have long been considered for their antibacterial capacity, although their cytotoxicity has significantly hindered their progress toward clinical application [50,51,52,53,54,55]. Meanwhile, titanium dioxide nanoparticles (TiO_2_NPs) have shown antibacterial efficacy while demonstrating better cytocompatibility [56,57,58,59,60] and even promoting bone formation owing their osteoconductive properties [57,61,62,63,64]. The particle’s surface area to volume determines the binding and ion release properties, which are crucial for antimicrobial activity. This relationship between size and reactivity (or antimicrobial activity) is inversely related. This potency is a metal ion release rate measure, where a higher relative surface area (and thus smaller size) allows for a greater ion release rate [43,44,50,65]. In the context of the medical application mentioned above, titanium is often the base material used as a substrate for these applications, with an ubiquitous presence in orthopedic surgery.

In order to take full advantage of the antibacterial activity of the aforementioned nanoparticles, one of the approaches is the deposition of a bioactive coating that imparts antibacterial and many other properties to the selected substrates [50,66,67,68,69,70]. While bioactive coating very often refers to the use of biocompatible polymers, functionalized or not, layer-by-layer (LbL) self-assembly using biocompatible polyelectrolytes has gained a lot of interest due to its versatility, cost effectiveness, and most importantly, for its ease of application on almost any substrate or surface, porous or dense, and with a two- or three-dimensional configuration [71,72,73]. This technique implies alternating applications of oppositely charged polyelectrolytes to form a multilayer (PEM) coating on a substrate. Through the selection of appropriate polyelectrolytes and their expected outcomes, this PEM can improve osteoblast adhesion and proliferation and reduce bacterial colonization and growth [72]. Chitosan and alginate, positively and negatively charged, respectively, are the two most commonly used polyelectrolytes for the LbL coating. They are abundant, naturally derived, non-toxic, and biocompatible, with innate antimicrobial and osteoconductive properties.

Therefore, the aim of this work was to introduce a bioactive coating using LbL deposition of chitosan and alginate with embedded TiO_2_-based nanoparticles on highly heterogeneous porous titanium substrates, mimicking the microstructure of commonly used orthopedic implants. The novelty of the proposed bioactive coating arises from the methodology used for the coating on a heterogenous porous substrate and a side-by-side study of silver and titanium oxide nanoparticles to demonstrate the overall superiority of TiO_2_NPs in terms of biocompatibility compared to AgNPs in such a bioactive coating. The hypothesis was that when TiO_2_ are encapsulated in the PEM made of chitosan and alginate, the coating will provide an effective antibacterial property by creating a concentration gradient and sustained release of mNP and will minimize the risk of antibiotic resistance while enhancing the osteogenic cell viability and proliferation on the coating. To verify this hypothesis, a thorough study was conducted to quantitatively assess the coating’s antibacterial activity and osteoblastic cell proliferation. First, we developed and characterized a PEM coating with embedded TiO_2_NPs and AgNPs (control) on porous titanium substrates. The in vitro viability and differentiation of MC3T3-E1 preosteoblast cells cultured on the coating as well as the antimicrobial activities of coated titanium substrates against *S. aureus* were assessed through a side-by-side comparison of TiO_2_NPs and AgNPs. Our goal was to establish a proof-of-concept for the use of these methods and formulations to guide advances toward more effective infection prevention and enhanced bone growth of orthopedic implants.

## 2. Materials and Methods

### 2.1. Materials

Titanium rods were purchased from Amplify Inc. (Scarborough, ME, USA). High-molecular-weight chitosan (>90% deacetylated) was purchased from MP Biomedicals (Solon, OH, USA). Alginic acid sodium salt (alginate) and silver nanopowder (<100 nm particle size, contains PVP as a dispersant) were purchased from Sigma Aldrich (catalog # 730815, Saint Louis, MO, USA). TiO_2_NP dispersion (rutile, 40 wt%, 30–50 nm, Stock #: 5484WJ) was purchased from Nanostructured and Amorphous Materials Inc. (Garland, TX, USA). Alpha MEM media without phenol red (αMEM), AlamarBlue, and fetal bovine serum (FBS) were purchased from Thermo Fisher Scientific (Waltham, MA, USA). Alpha-MEM cell culture medium with nucleosides and without ascorbic acid (αMEM+) was purchased from Invitrogen (Waltham, MA, USA). MC3T3-E1 murine preosteoblast cells and MSSA (DNC274, ATCC 29213) were purchased from ATCC (Manassas, VA, USA). An Alkaline Phosphatase Assay Kit (Colorimetric) was purchased from Abcam (Cambridge, UK). Luria–Bertani broth (LB Broth Miller) was purchased from BioShop (Burlington, ON, Canada).

### 2.2. Bacterial Strains

The standard strains of *S. aureus* DNC274 and ATCC 29213 were cultured on LB media for 24 h. A single colony was picked and cultured in LB media supplemented with 2% glycerol and stored at −80 °C until used for antimicrobial testing.

### 2.3. Titanium Substrate Preparation and Surface Functionalization

A cylindrical semi-porous 3D-printed titanium rod of 4.5 mm in diameter and 25 mm in length was used as the substrate (Figure 1). Titanium rods were cut transversely to produce approximately 3 mm thick disks. Coronally, one half of the substrate was solid titanium, while the other half had a porous microstructure with a 400 μm average pore size, a 300 µm strut diameter, and was approximately 65% porous. Prior to LbL deposition, chemical crosslinking was adapted from Martin et al. [74] to achieve surface functionalization by amine groups. All cut semi-porous titanium disks were washed three times, 10 min at a time, in acetone, ethanol, and then ultrapure water, and then blown dry with inert nitrogen gas. Disks were carefully submerged in piranha solution and stirred for 1 h to further clean and hydroxylate specimens. Disks were then removed from the solution and washed three times in ultrapure water. To produce amino-functionalized disks, samples were immersed in a 2% solution of (3-aminopropyl) triethoxysilane (APTES, Sigma Aldrich, Saint Louis, MO, USA) for 1 h. Samples were then washed five times with acetone to remove any residual silane groups. To facilitate and enable the deposition and crosslinking of the primary polymer layer, samples were then treated in 4% glutaraldehyde solution under stirring for 8 h. Samples were then removed and washed thrice in ultrapure water. The primary chitosan layer was applied by immersing samples in 0.1% chitosan solution for 8 h, and then rinsing non-adhered chitosan from the surface with ultrapure water.

### 2.4. TiO_2_ and Ag Nanoparticle Size Measurement

Prior to the experiments, the AgNPs and TiO_2_NPs were suspended in water and their size distribution was examined using the Nanosight NS300 Nanoparticle Tracking Analyzer (NTA, Salisbury, UK) to ensure that the purchased materials met the specifications on their labels. Following that, 1 mg/mL suspensions were prepared using water as the diluent and then diluted 500-fold to allow effective and accurate size measurements.

### 2.5. Preparation of mNP Suspension Prior to Encapsulation in LbL Coating

To prepare three different concentrations of alginate-TiO_2_NP suspensions, 40 wt% aqueous stock suspension of TiO_2_NPs (calculated to an equivalent of ≈670 mg/mL) was first diluted using ultrapure water to a concentration twice that of the final desired concentration of the most concentrated dispersion group. An aliquot of this initial stock suspension was diluted to a concentration of ≈55 mg/mL by adding 1.2 mL of stock suspension to ≈14 mL of ultrapure water. Then, 7 mL of 0.2% alginate and 1.75, 3.5, or 7 mL of the diluted TiO_2_NPs were transferred into three 15 mL tubes, mixed, and then filled with ultrapure water to a final volume of 14 mL, to achieve a final alginate concentration of 0.1% and TiO_2_NP concentrations of ≈7 ± 1, ≈ 14 ± 1, and ≈ 28 ± 1 mg/mL. Suspensions were vortexed vigorously for 1 min each to achieve uniform dispersions.

To prepare two different concentrations of alginate-AgNP suspensions, PVP-coated nanosilver powder was dispersed in ultrapure water at a concentration twice that of the highest intended AgNP group concentration. Stock dispersions were made to a concentration of 8 mg/mL. To ensure uniform dispersion, the solution was vortexed vigorously for 1 min, and then bath-ultrasonicated for 1 h. Meanwhile, 7 mL of 0.2% alginate was added to 2 separate 15 mL tubes. When ready, AgNP suspensions were added to the tubes containing alginate solutions, and filled to a final volume of 14 mL with ultrapure water, to obtain a final alginate concentration of 0.1% and AgNP concentrations of 0.4 and 4 mg/mL.

### 2.6. Preparation of the Polyelectrolytes for LbL Deposition

In this step, 0.2% chitosan solution was prepared by adding 20 mg/mL (0.2 g/100 mL) chitosan powder to 1% glacial acetic acid and stirring overnight until completely dissolved. Immediately prior to all experiments, aliquots of appropriate volume were isolated, and the pH was adjusted to 6.0 using 10 M and 1 M sodium hydroxide. The final concentration of the chitosan solution was then adjusted to 0.1% with ultrapure water.

Next, 0.2% alginate solution was prepared by adding 20 mg/mL (0.2 g/100 mL) sodium alginate powder to double-distilled water and stirred overnight. For experimental conditions using alginate without nanoparticles, aliquots were further diluted to a concentration of 0.1%.

### 2.7. mNP Embedding and LbL Coating Procedures

After the surface functionalization of the titanium substrate (Section 2.3), the subsequent coating of the multilayers was performed according to the protocol adapted from Zhong et al. [73]. Briefly, each polymer layer was applied similarly to the above layers, where the samples were immersed in 15 mL centrifuge tubes containing chitosan/alginate and rotated for 15 min to obtain a homogenous coating. After removal, the samples were washed twice in ultrapure water to remove nonadsorbed polymer from the sample surface. Samples were then immersed in the appropriate subsequent solution/suspension. This process was repeated until the desired number of bilayers was applied, at which point the disks were washed and air-dried overnight.

Samples coated with alginate containing ≈7 ± 1, ≈14 ± 1, and ≈28 ± 1 mg/mL of TiO_2_NPs were labeled 10 LbL^+^[TiO_2_NP]_l_, 10 LbL^+^[TiO_2_NP]_m_, and 10 LbL^+^[TiO_2_NP]_h,_, respectively. Samples coated with alginate containing 0.4 and 4 mg/mL of AgNPs were labeled 10 LbL^+^[AgNP]_l_ and 10 LbL^+^[AgNP]_h_, respectively. Samples coated using alginate without nanoparticle additives were labeled 10 LbL^−^NP, representing a PEM made of otherwise unmodified chitosan and alginate. Control samples, i.e., uncoated Ti substrate (or bare) were labeled B (Table 2).

### 2.8. Investigation of LbL Deposition Using Quartz Crystal Microgravimetry with Dissipation (QCM-D)

The in situ LbL build-up was determined using a QSense QCM-D Analyzer (Biolin Scientific Inc., Gothenburg, Sweden) instrument. To best mimic the surface properties of the semi-porous titanium implant samples that were used in all other experiments, QCM-D titanium crystals (QSX 310) (Biolin Scientific Inc., Gothenburg, Sweden) were employed as the substrate. However, in the chitosan and alginate LbL build-up, the polyelectrolyte solutions were run through the system on the bare Ti-based crystals without a prior surface functionalization, as the acid treatment could damage the Ti crystals.

Ti crystals were cleaned via UV–ozone (UV–ozone chamber Bioforce Nanosciences, Inc., Virginia Beach, VA, USA) treatment for 10 min, washed for 5 min in a 5:1:1 mixture of ultrapure water, 25% ammonia, and 30% hydrogen peroxide at 75 °C, followed by 10 min UV–ozone treatment. The experiment was set up to oscillate the crystals at their fundamental resonance frequency (*f* = 4.95 MHz), and their odd overtones (3–11) using electrodes supplying a radiofrequency voltage. The LbL process began with water flowing into the chambers at a rate of 400 μL/min for 5 min to establish a baseline measurement. Chitosan was then flowed in at the same rate for 3 min to ensure that the entire crystal was covered with the polymer. At this point, the flow pump was stopped for 15 min, to allow the polymer to adsorb onto the crystal surface. Water was then pumped for 3 min to remove nonadsorbed polymer. The tubing was switched to the alginate solution, and the same process was followed. The procedure was repeated until a total of five bilayers was applied. Frequency and dissipation measurements were performed in real-time using QSoft QCM-D software (version 3.0.10.286), while viscoelasticity and thickness calculations were performed using the Voigt-based viscoelastic model in the QTools software (version 3.0.10.286).

### 2.9. Analysis of Surface Morphology and Roughness of Coated Substrates Using Microscopy Techniques

The FEI Quanta450 Environmental Scanning Electron Microscope (ESEM) (FEI company, Hillsboro, OR, USA) was used to further confirm the deposition of the PEM, as well as to examine its porous microstructure before cell culture. The SEM was set to a full vacuum, and samples were lifted on the platform to a distance of 10 mm from the camera. Samples from each group were imaged at 5–10 kV, and final images were taken over 10 s for increased resolution.

Samples following MC3T3-E1 cell culture assays were also imaged with SEM for visualization of cell adhesion and spreading on the substrate surface. To prepare samples for this set of images, culture medium was removed from the wells, and samples were washed thrice with phosphate-buffered saline (PBS), before fixation using 4% paraformaldehyde for 1 h. Samples were then rinsed with PBS and processed for dehydration via immersion in graded concentrations of ethanol of 30%, 50%, 70%, 80%, 90%, and 100% for 15 min each. Subsequently, samples were dried using critical point drying and coated using a platinum sputter coater.

A MultiMode 8-HR AFM (Bruker, Billerica, MA, USA) was used to evaluate the surface roughness and morphology of non-functionalized bare titanium disks, disks coated with two bilayers or four bilayers, and those with TiO_2_NPs and without TiO_2_NPs. All samples were prepared following the same procedure as those for the cell culture and other experiments. PeakForce mode in air was used for all images, using a silicone probe with a spring constant k = 0.35 N/m and a resonance frequency *f*_0_ = 65 kHz. Images were acquired in 20 × 20 µm sections.

### 2.10. Cytotoxicity Assay

MC3T3-E1 cells (1 × 10^6^) were cultured in 10 mL of αMEM+ supplemented with 10% FBS and 1% penicillin/streptomycin in a T-75 flask. Every 2–3 days, the cell culture medium was refreshed until cells reached approximately 80% confluence, at which time adherent cells were collected from the surface of the flask, which was performed by removing the culture medium, gently washing with PBS, and then incubating for 5 min in 2 mL of 0.25% trypsin/EDTA. Following that, 5 mL of αMEM+ was added to the flask to resuspend the detached cells. The cells were inactivated with trypsin and were transferred to a 15 mL centrifuge tube. The cell density of the suspension was calculated using an automated cell counter to determine the volumes required for experimental seeding densities. Cells were centrifuged to attain a pellet and were resuspended with fresh αMEM-. The titanium disks were sterilized according to Holmes et al. [75]. Briefly, samples were immersed in 70% ethanol for 1 h, and further washed in a serial ethanol dilution of 35%, 17.5%, and 8.75% for 30 min. The disks were then washed three times for 10 min at a time in sterile 1 × PBS (pH 7.4) to remove any residual ethanol. After sterilization, specimens were placed in triplicate into wells of a 48-well microtiter plate. The wells without titanium disks placed inside were used as positive growth controls. Next, 400 μL MC3T3-E1 cells (3 × 10^4^ cells/mL) was then seeded and incubated for the predefined time points in a CO_2_-controlled incubator at 37 °C. αMEM- medium was refreshed every 3 days and 24 h prior to collection/testing time points for cell viability and differentiation assays.

The percent difference was used as a measure of relative cell viability of the experimental groups compared to control wells seeded with preosteoblasts without sample exposure. The calculation provided a quantitative description of how much more/less cell growth occurred in our experimental groups, compared to how much cell growth was observed in positive control wells, in order to determine the relative efficacy of the treatment.

### 2.11. MC3T3-E1 Viability Assessment with AlamarBlue

On days 1 (24 h after initial seeding), 4, 7, and 14, the cells were stored at −80 °C for later assessment of ALP activity. A fresh 400 μL of αMEM- medium containing 10% AlamarBlue (9:1 ratio between cell culture medium and AlamarBlue) was added to the wells and incubated for 4 h in the dark. After incubation, 100 μL from each well was withdrawn in triplicate and added to a 96-well clear flat-bottomed UV-transparent microplate (Sigma Aldrich, Saint Louis, MO, USA). The wells of the 48-well plate were once again replenished with 400 μL of αMEM- and returned to the incubator. A Spectramax i3 spectrophotometer (Molecular Devices, San Jose, CA, USA) was used to measure the absorbance values of the media samples at 570 nm and 600 nm. The percentage difference in AlamarBlue reduction was calculated from the absorbance data and the extinction coefficients of resazurin according to the protocol provided by the manufacturer.

### 2.12. Assessment of MC3T3-E1 Osteogenic Differentiation via ALP Activity Analysis

On days 1, 4, 7, and 14 of cell culture with coated disks, αMEM- cell culture medium was extracted from each well, and alkaline phosphatase (ALP) activity assays were performed on the above-mentioned media extracts according to the ab83369 Alkaline Phosphatase Assay Kit (Colorimetric) manual. Briefly, a standard curve was generated prior to assaying the experimental samples. Then, 80 µL of each sample was poured into each well of a 96-well microtiter plate, 80 µL of media control group, and 120 µL of each standard dilution. Furthermore, 20 µL of Stop Solution was added to the control wells, along with 50 µL of 5 mM para-nitrophenyl phosphate (pNPP) to the sample and control wells. Meanwhile, 10 µL of ALP enzyme was added to each of the standard dilution wells. Plates were covered with foil to protect from light and incubated at 25 °C for 60 min, followed by the addition of 20 µL of Stop Solution to the sample and standard wells. The plates were gently vortexed, and colorimetric measurements were performed using a Spectramax i3 spectrophotometer (Molecular Devices, San Jose, CA, USA) at 405 nm.

### 2.13. Antimicrobial Assessment of Coated Ti Substrates

*S. aureus* (DNC274, ATCC 29213) strains were grown on LB agar plates from the glycerol stocks. A single colony was transferred to 5 mL LB media and incubated at 37 °C for 6 h, further subcultured in 15 mL LB media with OD_600_ 0.05, and then incubated overnight at 37 °C and 200 rpm. The next day, the cultures were centrifuged to remove the media, and the pellet was washed twice with 1 × PBS and resuspended in LB media. The OD was adjusted to 0.05 OD (~1 × 10^4^ CFU/mL). The coated titanium disks along with the appropriate controls were placed in a 48-well microtiter plate, seeded with 400 µL of *S. aureus* suspensions at 1 × 10^4^ CFU/mL, and incubated at 37 °C for 24 h without shaking. After 24 h of incubation, the planktonic bacterial growth was determined by removing 100 µL of suspension from each sample well and transferring it in triplicate to a 96-well microtiter plate. These samples were serially diluted 10-fold and plated on LB agar, then incubated overnight at 37 °C, and the CFUs were determined via colony counting. The cells in the 48-well microtiter plates were removed and gently washed thrice with 1 × PBS, and titanium disks were transferred to 1.5 mL Eppendorf tubes containing 1 mL of 1 × PBS. These tubes were then vortexed at high intensity for 1 min to detach adhered bacteria and resuspend them into 1 × PBS. These samples were further serially diluted and plated, and colony counts were determined as CFU.

### 2.14. Statistical Analysis

Statistical comparison between samples was performed via two-way analysis of variance (ANOVA). Tukey’s post hoc comparison was performed in Prism5. Values are means from at least 3 parallels. Bars are standard deviations (SDs). Differences were considered statistically highly significant at *p* < 0.01.

## 3. Results and Discussion

### 3.1. Nanoparticle Characterization

**Figure 2:**
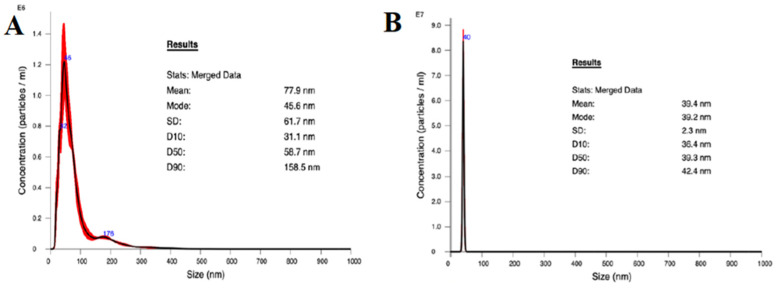
Nanoparticle size distribution as measured using NTA. (**A**) TiO_2_NP and (**B**) AgNP.

The average size of the AgNPs was 77.9 nm, and 50% were below 100 nm as determined by NTA (Figure 2A). Although the mean was higher than intended, likely due to some aggregation producing larger particles, the distribution of particle sizes was effectively unimodal at about 45.6 nm. These results support the method used to suspend the purchased AgNPs. The distribution of TiO_2_NP sizes (Figure 2B) from the purchased nanoparticle suspension had a narrow unimodal peak at 39.2 nm, and an average diameter of 39.4 nm, which is consistent with the description from the manufacturer of rutile TiO_2_NPs.

### 3.2. Validation of LbL Deposition Using QCM-D and PEM Coating Characterization

**Figure 3:**
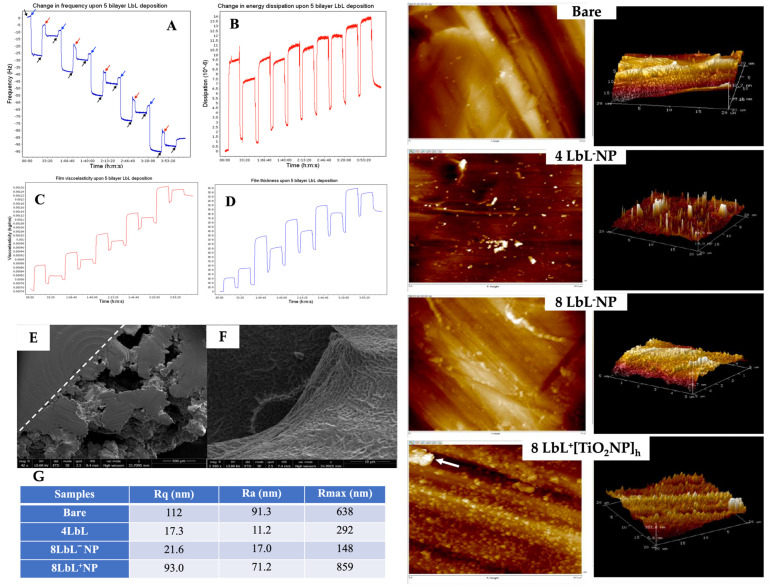
Panels (**A**–**D**): QCM-D plot of (**A**) the change in frequency (Hz) overtone of a titanium-oxide-coated quartz crystal undergoing LbL application of 5 bilayers of chitosan and alginate polyelectrolytes. Arrows denote time points at which specific solutions were applied. Black—ultrapure water, blue—chitosan, red—alginate; (**B**) change in dissipation; (**C**) changes in viscoelastic properties; and (**D**) change in film thickness over the coating period. Panels (**E**,**F**): SEM images capturing (**E**) top-down view of semi-porous titanium showing the structure’s solid and porous sides of the PEM-coated titanium, and (**F**) close-up view into a pore of a sample coated with chitosan and alginate shows successful PEM application and penetration into the pore. The two panels on the right show typical 2D and 3D AFM images of bare titanium substrate, with 4 and 8 LbL^−^NP; ^+^[TiO_2_NPs]_h_ (≈28 mg/mL TiO_2_NPs: arrow denotes a potential agglomeration of TiO_2_NPs). Panel (**G**): Surface roughness for different titanium substrates as determined using AFM. Scale bars vary with the images.

To validate the LbL self-assembly of the PEM, its simulated in situ build-up was achieved using QCM-D. The frequency decreased linearly during the LbL process, indicating the consistent sequential build-up of the PEM on the titanium dioxide quartz crystal (Figure 3A). Dissipation measurements followed the frequency changes (Figure 3B), while viscoelasticity and thickness increased stepwise with each bilayer (Figure 3C,D, respectively). We can see from the thickness plot (Figure 3D) that at the first and last water flow stages, the thinnest layer had a thickness of 5.4982 × 10^−14^ m (≈0.05 picometer) and reached a thickness of 1.8529 × 10^−8^ m (≈18.5 nm) after deposition of eight layers of polyelectrolytes. This final PEM was thinner than anticipated, possibly due to the flow rate. A previous study indicated an increase in the thickness of PME when the water flowed at 100 µL/min compared to 400 µL/min [76]. The frequency change immediately after the washing step also showed a sharp increase, which could be associated with some loss of polymer electrolytes, potentially destabilizing the subsequent polymer depositions, further explaining the formation of a thinner coating in situ. A thicker PME deposition can be expected for the functionalized titanium substrates used for all other experiments, as the absence of surface functionalization could have contributed to reducing adsorption of the initial layer.

Examples of the electron microscopy micrographs of coated titanium substrates are shown in Figure 3E,F. Using a dotted line as a visual aid, the top-down view of the sample in Figure 3E denotes the structural distinction between the solid and porous sides of the substrate. These images demonstrate the successful coating of the titanium substrates with the PEM. The porous structure created by the PEM has been reported to promote bone ingrowth [43,44].

The QCM-D results for PEM were confirmed via AFM analysis. The AFM images showed that the application of PEM smoothed the surface of the substrates, as a decrease in surface roughness was observed following the deposition of PEM onto the titanium substrates compared to the bare group (Figure 3 right panels and Figure 3G). This was particularly evident when considering the relative smoothing of the surface upon multilayer adsorption, where the comparatively high average roughness in the bare sample (Ra = 91.3 nm) was no longer reflected in samples with only 4 LbL (Ra = 11.2 nm). The subsequent increase in Ra after additional PEM deposition, i.e., 8 LbL (17.0 nm), although no significant, could be attributable to an increase in the porosity of the multilayer film [55]. As expected, the surface roughness increased when the TiO_2_NPs were embedded in the PEM coating (17.0 nm for 8 LbL^−^NPs versus 71.2 nm for 8 LbL^+^[TiO_2_NPs]_h_). The results indicated that the nanoparticles were within the appropriate size range and sufficiently monodispersed to evenly cover the entire substrate (including within the porous titanium lattice) with the PEM. Although this was a rare occurrence, the large peak on the AFM image of 8 LbL^+^[TiO_2_NPs]_h_ denoted with an arrow indicated that titanium nanoparticles might undergo aggregation. Overall, this increase in surface roughness after the encapsulation on the NPs is a beneficial characteristic, as it has been shown that cell adhesion and bone–implant contact are improved with greater roughness [45,76].

### 3.3. MC3T3-E1 Viability and Proliferation on PEM-Coated Ti Substrates

**Figure 4:**
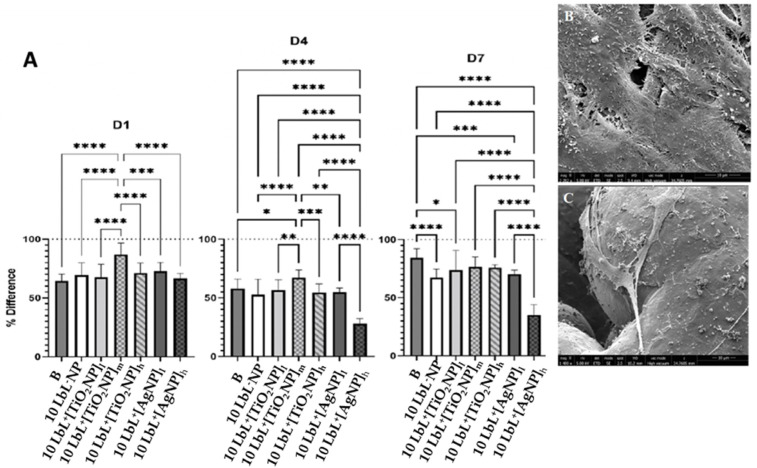
Panel (**A**) are the plots of AlamarBlue tests showing percent difference across test days. D1—after 1 day, D4—after 4 days, D7—after 7 days. Dotted line at y = 100 refers to the measured AlamarBlue results of the reference group (cells only, no samples). Stars denote significant differences (*n* = 3), *—<0.05; **—<0.01; ***—<0.001; ****—<0.0001. Group labels are defined as 10 LbL^−^NP—with 5 bilayers of chitosan/alginate without NP, 10 LbL^+^[TiO_2_NP]_l_—with 5 bilayers of chitosan/alginate with 7 mg/mL TiO2NP, 10 LbL^+^[TiO_2_NP]_m_—with 5 bilayers of chitosan/alginate with 14 mg/mL TiO2NP, 10 LbL^+^[TiO_2_NP]_h_—with 5 bilayers of chitosan/alginate with 28 mg/mL TiO2NP, 10 LbL^+^[AgNP]_l_—with 5 bilayers of chitosan/alginate with 0.4 mg/mL AgNP, 10 LbL^+^[AgNP]_h_—with 5 bilayers of chitosan/alginate with 4 mg/mL AgNP, B—uncoated Ti substrate (Bare). (**B**,**C**) SEM images of titanium substrate surfaces showing MC3T3-E1 adhesion and spreading on 10 LbL^+^[AgNP]_h_ on the solid and porous parts of the substrate (scale bar: 10 µm).

The viability of preosteoblast MC3T3-E1 cells on LbL^+^ TiO_2_NP was determined at the 1, 4, and 7-day time points with AlamarBlue using LbL^−^NP as the negative control and uncoated samples along with LbL-coated embedding AgNPs as positive controls (Figure 4A). This analysis showed significant differences in cell viability among various time points (*n* = 3) (*n* = 3; *p* < 0.0001) and groups (*n* = 3; *p* < 0.0001) as well as between the two variants when combined (*n* = 3; *p* < 0.0001). Coating significantly affected the viability of MC3T3-E1 cells at a given time and across days. Data were also categorized by the day of testing, as shown in Figure 4A. After day 1, 10 LbL^+^[TiO_2_NP]_m_ samples showed higher cell viability than all other test groups (*n* = 3; *p* < 0.0003), while the others performed similarly. On day 4, these same cells maintained their superior viability (*n* = 3; *p* < 0.05) compared to all other groups. As expected, cells exposed to 10 LbL^+^[AgNP]_h_ experienced a reduction in relative viability, resulting in these showing less viability than all other groups (*n* = 3; *p* < 0.0001). At day 7, regardless the titanium oxide NP concentration used, there was no significant difference among these samples; the most significant remaining difference between the groups was the sustained reduction in viability in 10 LbL^+^[AgNP]_h_, which was still lower than all other groups (*n* = 3; *p* < 0.0001). This is a very interesting finding considering that a much higher concentration of titanium oxide NPs was used compared to AgNPs for cell viability, confirming the much lower cytotoxicity of titanium NPs to osteoblastic cells.

**Figure 5:**
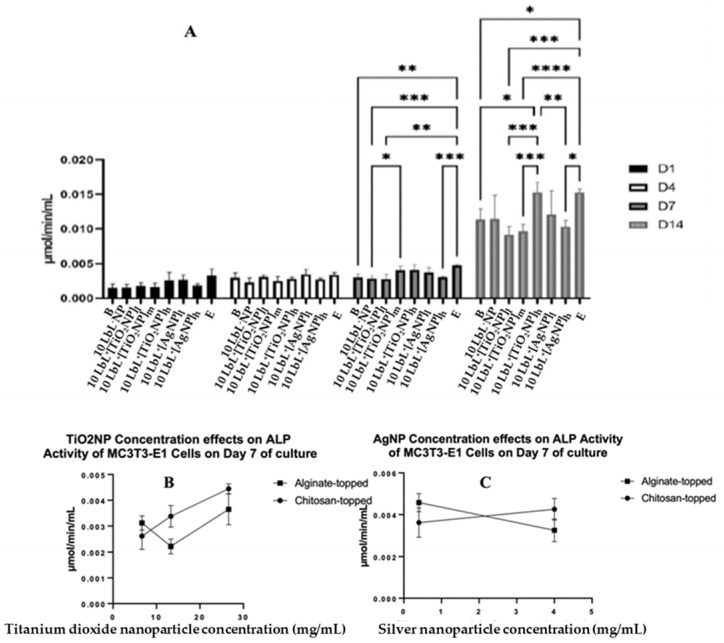
(**A**) ALP activity of coated samples across test days where different top-coated groups are combined. Stars denote significant differences (*n* = 3) *—<0.05, **—<0.01, ***—<0.001, ****—<0.0001. Group labels are defined as 10 LbL^−^NP—with 5 bilayers of chitosan/alginate without NP, 10 LbL^+^[TiO_2_NP]_l_—with 5 bilayers of chitosan/alginate with 7 mg/mL TiO_2_NP, 10 LbL^+^[TiO_2_NP]_m_—with 5 bilayers of chitosan/alginate with 14 mg/mL TiO_2_NP, 10 LbL^+^[TiO_2_NP]_h_—with 5 bilayers of chitosan/alginate with 28 mg/mL TiO_2_NP, 10 LbL^+^[AgNP]_l_—with 5 bilayers of chitosan/alginate with 0.4 mg/mL AgNP, 10 LbL^+^[AgNP]_h_—with 5 bilayers of chitosan/alginate with 4mg/mL AgNP, B—uncoated Ti substrate (Bare), and E—cells only. Panels (**B**,**C**): Linear regression plots of nanoparticle concentration’s effect on ALP activity of MC3T3-E1 cells exposed to nanoparticle-embedded PEMs.

To determine whether the presence of NPs affects the MC3T3-E1 cells’ osteogenic differentiation, the ALP activity was assessed on extracted cell culture media to determine the extent of preosteoblast differentiation using alkaline phosphatase as a biomarker (Figure 5A). In these assays, groups were also separated based on the polymer layer applied as the uppermost coating: nanoparticle-incorporated alginate or chitosan. The results were consistent with the previous literature [46,47]. Despite a significant effect of the top-coat on day 7 (*n* = 3; *p* = 0.0001), this difference was not seen on any other day tested, indicating that the top coat was not a significant factor in the ALP activity of cells exposed to samples throughout the experimental period. Therefore, MC3T3-E1 analyses were conducted with those groups combined. A two-way ANOVA was performed on the combined ALP data, and it was found that there was a main effect of day (*n* = 3; *p* < 0.0001), of group (*n* = 3; *p* < 0.0001), and an interaction of group × day (*n* = 3; *p* < 0.0001). There were no statistical differences between the groups on days 1 and 4. On day 7, cells exposed to 10 LbL^+^[TiO_2_NP]_h_ samples showed slightly higher ALP activity compared to those exposed to 10 LbL^+^[TiO_2_NP]_l_ (*n* = 3; *p* = 0.0453). Tests on day 14 showed a significant increase in ALP activity in all groups compared to their respective counterparts on previous days. On day 14, cells exposed to 10 LbL^+^[AgNP]_l_ showed one of the highest absolute means of ALP activity (0.01528 µmol/min/mL), second only to the cell-only control group (0.01530 µmol/min/mL). This increased ALP activity was greater than those of the groups without nanoparticles (*n* = 3; *p* < 0.05) and with 10 LbL^+^[TiO_2_NP] and 10 LbL^+^[TiO_2_NP]_h_ (*n* = 3; *p* < 0.001), but not statistically different from those exposed to 10 LbL^+^[TiO_2_NP]_l_ and 10 LbL^+^[AgNP]_h_.

A linear regression was performed on an isolated data segment, focusing on the potential nanoparticle dose-dependent responses that cells may have to the construct. These data are presented in Figure 5B,C. Here, a difference in cell responses was observed depending on the nanoparticle concentration incorporated into the PEM. For TiO_2_NP-containing samples, a significant positive relationship between nanoparticle concentration and ALP activity was found in the chitosan-topped group (R^2^ = 0.0832, F(1,7) = 35.74, *n* = 3; *p* = 0.0006) but not the alginate-topped group. In AgNP-containing samples, a negative relationship was observed between concentration and ALP activity (R^2^ = 0.7328, F(1,4) = 10.97, *p* = 0.0296) in the alginate group. This suggests that PEMs with higher concentrations of TiO2NPs, especially when coated with chitosan, may promote greater ALP activity, while alginate-coated PEMs with increasing concentrations of AgNPs show decreasing ALP activity in response.

### 3.4. Antimicrobial Activity

**Figure 6:**
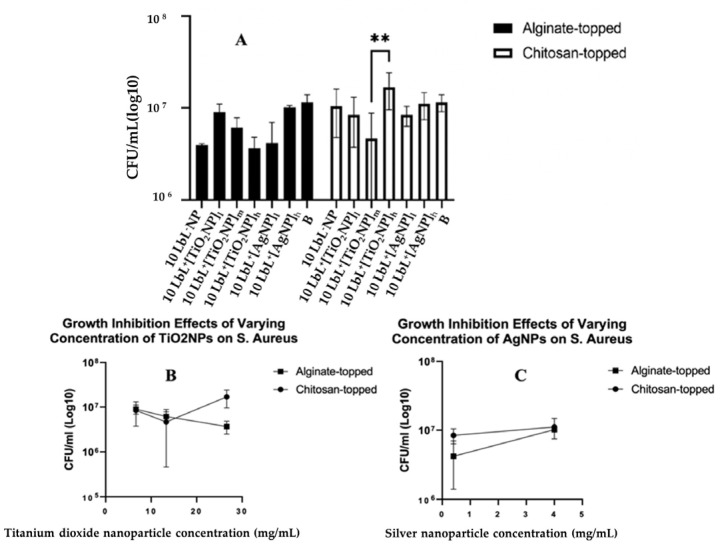
Panel (**A**): Estimated bacterial growth based on cell counts of adherent bacteria exposed to samples in liquid media for 24 h. Stars denote significant differences (*n* = 3), **—<0.01. Group labels are defined as 10 LbL^−^NP—with 5 bilayers of chitosan/alginate without NP; 10 LbL^+^[TiO_2_NP]_l_—with 5 bilayers of chitosan/alginate with 7 mg/mL TiO_2_NP; 10 LbL^+^[TiO_2_NP]_m_—with 5 bilayers of chitosan/alginate with 14 mg/mL TiO_2_NP; 10 LbL^+^[TiO_2_NP]_h_—with 5 bilayers of chitosan/alginate with 28 mg/mL TiO_2_NP; 10 LbL^+^[AgNP]_l_—with 5 bilayers of chitosan/alginate with 0.4 mg/mL AgN; 10 LbL^+^[AgNP]_h_—with 5 bilayers of chitosan/alginate with 4mg/mL AgNP; B—uncoated Ti substrate (Bare). Panels (**B**,**C**) shows linear regression plots of nanoparticle concentration’s effect on antibacterial activity against *S. aureus* cells exposed to nanoparticle-embedded PEMs.

The antimicrobial efficacies of the coated samples, tested on a mature 24 h biofilm of *S. aureus* and CFU, were determined. Bonferroni’s multiple comparisons showed a distinct difference in the 10 LbL^+^[TiO_2_NP]_h_ topmost polymer layer (*n* = 3; *p* = 0.0007) and chitosan-topped 10 LbL^+^[TiO_2_NP]_m_ and 10 LbL^+^[TiO_2_NP]_h_ (*n* = 3; *p* = 0.0041) (Figure 6A).

No significant statistical differences between nanoparticle types were observed. In the interest of understanding the potential dose–response characteristics of the antibacterial activity, nanoparticle-containing groups were isolated into their respective datasets, and a simple linear regression was performed on each of them (Figure 6A). For samples containing TiO_2_NPs, a significant negative relationship was found between nanoparticle concentration and CFU in the alginate-topped group (R^2^ = 0.6798, F(1,7) = 14.86, *n* = 3; *p* = 0.0063) but no significance in the chitosan-topped group. Surface charge affects protein absorption, cell adhesion, and proliferation. Positive charges can promote cell spreading, proliferation, and immune system signaling, leading to regenerative responses and better biocompatibility [48,77]. Surface charge density along with two primary forces, i.e., van der Waals and electrostatic interactions, also impact bacterial adhesion. Bacteria typically carry a net negative charge, resulting in greater adhesion to positively charged surfaces [49]; however, the bacterial adhesion species-specific *S. aureus* demonstrated higher adhesion on the cationic surfaces [51]. In AgNP-containing samples, a positive relationship was observed between concentration and bacterial growth (R^2^ = 0.7667, F(1,4) = 13.14, *p* = 0.0222) in the alginate group. These data suggest a trend toward dose-dependent antibacterial activity in alginate-topped TiO_2_NP-incorporated PEMs, although the magnitude of growth inhibition was less than anticipated. One source of this issue could be that the size of the wells in the 48-well culture plate in which the samples were cultured with the suspended bacteria was larger than the sample size. This could have limited the bacteria’s exposure to the coating, as they could have grown in the liquid culture without direct contact with the coating. This could have given the bacteria the opportunity to multiply to a number that would be unmanageable by the coating. Another reason for observing such a result could be related to the seeding density and the culture materials used. Other studies have been conducted using widely varying methods and seeding densities (from 10^5^ CFU/mL [52] to 10^6^ CFU/mL [53], to 10^9^ CFU/mL [54]).

## 4. Conclusions

Novel and more effective approaches for preventing bacterial seeding onto orthopedic implants are of paramount interest. This research sought to establish a proof-of-concept for a novel bacteriostatic, bactericidal, and osteoconductive surface coating for use as an anti-infective surface modification and preventative measure against PJI. The LbL technique was used to assemble PEMs with embedded nanoparticles of titanium dioxide or silver on the titanium substrate featuring both solid and porous matrices. Several surface characterization techniques confirmed that the PEM and its embedded nanoparticles were successfully deposited on the substrate. The PEM-embedded TiO_2_NPs showed superior preosteoblast cell viability even at higher doses of TiO_2_NPs as well as a promoted osteogenic differentiation of osteoblastic cells compared to PME-embedded AgNPs. The antibacterial activity was found to be similar for PMEs whether TiO_2_ or AgNPs was embedded in PMEs. However, a dose-dependent antibacterial activity trend toward growth inhibition was observed for tested concentrations of TiO_2_NPs.

Although further in vitro and in vivo studies are necessary to refine the PEM’s coating and to perform more quantitative analyses of coating embedding titanium oxide capabilities and dose-dependent cell viability, proliferation, and osteogenic differentiation, the findings reported here are considered a first step toward developing a suitable coating for encapsulation of TiO_2_ that has the desired antibacterial effect but does not substantially harm the host cell. Such a coating may be an alternative to the use of highly toxic AgNPs for the purposes of infection prevention and enhanced bone growth. Regarding the feasibility for clinical translation, it should be noted that coating procedure can be automated (Automated Spray LbL technology or Automated Spin-Assisted LbL Assembly) and integrated into the manufacturing process in an aseptic condition as the last step before the packaging of the implant.

## Figures and Tables

**Table 1:** Antimicrobial activity of metal nanoparticles.

Nanoparticles	Microorganism	References
TiO_2_ nanoparticles	*Staphylococcus aureus*, *Escherichia coli*, *Psedomonas aeruginosa*, *Proteus vulgaris*	[39,40,41,42]
Ag nanoparticles	*E. coli*, *Pseudomonas* species, *Staphylococci* species, *Candida* species, *Bacillus* species	[22,23,24,25]
Au nanoparticles	*E. coli*, *S. aureus*, *Klebsiella pneumoniae*, *Candia* species	[35,36,37,38]
ZnO nanoparticles	*S. aureus*, *E. coli*, *Candia albicans*, *P. aerugnosa*, *Proteus mirabilis*	[26,27,28,29]
CuO nanoparticles	*E. coli*, *S. aureus*, *P. aeruginosa*, *Candida* species, *bacillus subtilis*	[30,31,32,33,34]

**Table 2:** Samples’ descriptions and abbreviations.

Sample Description	Experiments	Abbreviation
Uncoated Ti substrate (bare)	AFM/QCM-D	**B**
With 2 bilayer of Chitosan/Alginate no nanoparticles (NP)	AFM/ QCM-D	**4 LbL^−^NP**
With 4 bilayer of Chitosan/Alginate no NP	AFM/ QCM-D	**8 LbL^−^NP**
With 4 bilayer of Chitosan/Alginate with 28 mg/mL TiO_2_ NP	AFM/ QCM-D	**8 LbL^+^[TiO2NP]_L_**
With 5 bilayer of Chitosan/Alginate no NP	Viability/ALP/Antimicrobial assay	**10 LbL^−^NP**
With 5 bilayer of Chitosan/Alginate with 7 mg/mL TiO_2_NP [low]	Viability/ALP/Antimicrobial assay	**10 LbL^+^[TiO_2_NP]_l_**
With 5 bilayer of Chitosan/Alginate with 14 mg/mL TiO_2_NP [medium]	Viability/ALP/Antimicrobial assay	**10 LbL^+^[TiO_2_NP]_m_**
With 5 bilayer Chitosan/Alginate with 28 mg/mL TiO_2_NP [high]	Viability/ALP/Antimicrobial assay	**10 LbL^+^[TiO_2_NP]_h_**
With 5 bilayer Chitosan/Alginate with 0.4 mg/mL AgNP	Viability/ALP/Antimicrobial assay	**10 LbL^+^[AgNP]_l_**
With 5 bilayer Chitosan/Alginate with 4 mg/mL AgNP	Viability/ALP/Antimicrobial assay	**10 LbL^+^[AgNP]_h_**

## Data Availability

The data presented in this study are available on request from the corresponding author.
